# miR-155 is positively regulated by CBX7 in mouse embryonic fibroblasts and colon carcinomas, and targets the KRAS oncogene

**DOI:** 10.1186/s12885-017-3158-z

**Published:** 2017-03-04

**Authors:** Floriana Forzati, Marco De Martino, Francesco Esposito, Romina Sepe, Simona Pellecchia, Umberto Malapelle, Gianluca Pellino, Claudio Arra, Alfredo Fusco

**Affiliations:** 1 Istituto di Endocrinologia ed Oncologia Sperimentale “G. Salvatore” - CNR c/o Dipartimento di Medicina Molecolare e Biotecnologie Mediche, Università degli Studi di Napoli “Federico II”, Naples, Italy; 2 Dipartimento di Sanità Pubblica, Università degli Studi di Napoli “Federico II”, Naples, Italy; 3 Unità di Chirurgia Colorettale, Dipartimento di Scienze Mediche, Chirurgiche, Neurologiche e dell’Invecchiamento, Seconda Università di Napoli, Naples, Italy; 4 Istituto Nazionale dei Tumori, Fondazione Pascale, Naples, Italy

**Keywords:** CBX7, miR-155, KRAS, Colon carcinoma

## Abstract

**Background:**

Loss of *CBX7* expression has been described in several malignant neoplasias, including human colon and thyroid carcinomas proposing *CBX7* as a tumor suppressor gene with a key role in cancer progression. This role is supported from the development of benign and malignant neoplasias in *Cbx7* null mice.

The aim of our work has been to investigate the mechanisms underlying the CBX7 oncosuppressor activity by analyzing the microRNAs (miRNAs) regulated by CBX7.

**Methods:**

The miRNA expression profiles of the mouse embryonic fibroblasts (MEFs) null for *Cbx7* and the wild-type counterpart were analyzed by the miRNACHIP microarray and then validated by qRT-PCR. To asses KRAS as target of miR-155 we evaluated the protein levels after transfection of the synthetic miR-155. Human colon carcinoma samples have been investigated for the expression of CBX7 and miR-155.

**Results:**

Twenty miRNAs were found upregulated and nine, including miR-155, downregulated in *cbx7-*null MEFS in comparison with the wild-type ones. Then, we focused on miR-155 since several studies have shown its deregulated expression in several human malignancies and, moreover, was the most downregulated miRNA. Subsequently, we searched for miR-155 target genes demonstrating that KRAS protein levels are directly modulated by miR-155. A direct significant correlation (*r* = 0.6779) between CBX7 and miR-155 expression levels was found in a set of human colon carcinoma tissue samples.

**Conclusion:**

miR-155 is positively regulated by CBX7 in MEFs and colon carcinomas, and has KRAS as one of the target genes likely accounting for the anti-apoptotic activity ascribed to miR-155 in some tissue contexts.

**Electronic supplementary material:**

The online version of this article (doi:10.1186/s12885-017-3158-z) contains supplementary material, which is available to authorized users.

## Background

CBX7 belongs to the polycomb repressive complex 1 (PRC1), a multiprotein complex that together with the polycomb repressive complex 2 (PRC2) inhibits the transcription of the developmental genes [1–3]. A crucial activity of CBX7 in tumor progression is supported by several studies [4]. Indeed, a drastic downregulation of CBX7 expression has been described in thyroid [5], pancreatic [6], colon [7], lung [8], gastric [9], bladder [10], breast [11] carcinomas, and a more advanced stage of neoplastic disease and a poor survival has been directly correlated to the loss of *CBX7* expression [6, 7]. Furthermore, when *CBX7* expression is restored in thyroid [5], gastric [9] and colon [7] carcinoma cells there is a decreased proliferation rate with the accumulation of the cells in the G1 phase of the cell cycle, suggesting a negative role of CBX7 on the control of cell growth and, particularly, in the regulation of the G1/S switch of the cell cycle [5].

CBX7 is able to interact with different proteins, modulating in positive or negative the expression of several genes implicated in various biological functions [4]. In particular, it positively regulates the expression of E-cadherin [12] that is required to maintain the regular morphology of epithelial cell, and whose loss of expression is associated with the epithelial-mesenchymal transition [13, 14]. The CBX7 activation of E-cadherin expression is caused by the interaction with histone deacetylase 2 and inhibition of its action on the CDH1 promoter [12]. Consistently, two studies, in thyroid [12] and pancreatic [6] carcinomas, report a direct correlation between the *CBX7* expression and the E-cadherin levels.

Moreover, we have also shown that CBX7 counteracts the HMGA-induced activation of the *SPP1* gene [15], encoding the chemokine osteopontin, that is highly overexpressed in several human carcinomas and has a key function in malignant transformation. Furthermore, CBX7 inhibits, by a similar mechanism, the promoter activity of Cyclin E gene [8], that enhances the transition of the G1 to S phase of the cell cycle then increasing the cell proliferation rate. Therefore, on the basis of all these studies, the absence of the *CBX7* gene expression plays an important function in the late stages of human malignancies [4].

MicroRNAs (miRNAs) have become known as a significant class of short endogenous RNAs that control gene expression at post-transcriptional level through base-pairing with their target mRNAs for direct cleavage or by inhibiting mRNA translation [16–18]. They have a central function in a lot of biological pathways, as developmental process, signaling transduction, stem cell differentiation, cell growth, and cancer [19, 20].

In the current study, we have carried out an analysis of miRNA expression profiling in mouse embryonic fibroblasts (MEFs) obtained from *Cbx7*-knockout (KO) and wild-type (WT) mice to search for the miRNAs modulated by the Cbx7 protein. Among the differentially expressed miRNAs in WT and *Cbx7*-null MEFs, we concentrated our attention on the miR-155, downregulated in homozygous *Cbx7*-KO MEFs in comparison to the WT. Consistently, we report downregulation of both *CBX7* and miR-155 expression in a set of colon carcinomas. Finally, we demonstrate that *KRAS* gene is a target of miR-155.

## Methods

### Cell cultures

Primary MEFs from *Cbx7* ^+/+^, *Cbx7* ^+/−^, *Cbx7* ^−/−^ and transgenic (TG) *Cbx7* mice, were obtained and grown as previously described [8].

Human embryonic kidney HEK 293 cells and lung cancer cell lines A549 were cultured as reported elsewhere [8, 21].

### miRNACHIP microarray

RNA labeling, hybridization on miRNA microarray chips and microarray analyses were carried out as reported elsewhere [22, 23].

Briefly, 5 μg of total RNA from each sample were biotin-labeled through reverse transcription by random examers. Hybridization was performed on a miRNA microarray chip [22] containing 368 probes in triplicate. Hybridization signals were identified through biotin binding of a streptavidin-alexa 647 conjugate by a Perkin-Elmer ScanArray XL5K (Perkin-Elmer, Wellesley, MA, USA) and quantified by the Quantarray software (Perkin-Elmer). Raw data were normalized and analyzed by GENESPRING software (Silicon Genetics, Redwood City, CA, USA). miRNAs were quantified by class comparison using Student’s *t* test procedure. Each sample was studied for miRNA expression profile in triplicate.

### Bioinformatic prediction of miRNA target genes

Genes potentially targeted by the selected miRNAs were found through on-line accessible tools i.e. miRanda (www.microrna.org), TargetScan (www.targetscan.org), or miRwalk (http://zmf.umm.uni-heidelberg.de/apps/zmf/mirwalk2)

### RNA extraction, reverse transcription and quantitative real time (qRT-PCR)

Total RNA was extracted from cells and tissue samples using Trizol (Invitrogen, Carlsbad, CA), according to manufacturer’s instructions. In particular, tissue samples were dissociated with homogenizer. For mRNA detection, 1 mg of RNA from each sample was reverse-transcribed using QuantiTech Reverse Transcription kit (Qiagen, Valencia, CA) [24] and then Real-Time PCR was performed by using iQ SybrGreen SuperMix (BioRad, Hercules, CA). qRT-PCR analyses were performed using the following primers:

- h CBX7-fw 5’-cgagtatctggtgaagtggaaa-3’
- h CBX7 rev 5’-gggggtccaagatgtgct-3’
- h KRAS-fw 5’-aggctcaggacttagcaagaa-3’
- h KRAS-rev 5’-gaaggcatcatcaacaccct-3’
- m KRAS-fw 5’- tgtggatgagtatgaccctacg-3’
- m KRAS-rev 5’- ccctcattgcactgtactcct-3’

For miRNA detection, RNA from each sample was reverse-transcribed using miScript reverse transcription Kit (Qiagen, Valencia, CA). Real-Time PCR was performed according to miScript System Kits (Qiagen, Valencia, CA) instructions [24]. Real-Time PCR reactions contained miScript Primer Sets (Qiagen, Valencia, CA), specific for mir-155 (5’-UUAAUGCUAAUCGUGAU AGGGGU-3’) and U6 (cod. MS00033740) (used to normalize RNA levels).

### Plasmids and transfections

miRNA transfection were performed as described elsewhere [23].

The *KRAS* 3’-UTR region, containing binding sites for miR-155, was amplified by PCR using the primers KRAS fw 5’-aatttctagaggcatactagtacaagtggt-3’ and KRAS rev 5’-aatttctagacagggatgacaaactatagg-3’. The amplified fragments were cloned as previously described [23]. Mutation of miR155-binding site (a**cgtaa**a) was synthesized by IDT (TEMA ricerca, Bologna, Italy).

### Protein extraction, western blotting, and antibodies

Protein extraction and Western blotting were performed as previously described [8]. Primary antibodies used were anti-KRAS (H00003845-M01, Abnova and sc-30, Santa Cruz), anti-Actin (sc-1616, Santa Cruz) anti-Gapdh (sc-32233, Santa Cruz). Western blotting detection reagents (Thermo Scientific, Rockville, IL) were used to visualize immunoblots.

### Dual-luciferase reporter assay

4 × 10^4^ 293 cells were co-transfected in 24-well dishes with the pGL3-*KRAS 3* **’**-UTR luciferase reporter construct or with the mutated *KRAS* 3**’**-UTR luciferase vector, together with the Renilla luciferase plasmid and with the RNA oligonucleotides (Ambion), using lipofectamine plus (Ambion). The pRL-TK control vector expressing Renilla luciferase (Promega) was used to normalize cell number and transfection efficiency. Luciferase activity was measured as previously reported [25].

### Collection and processing of surgical resections

Carcinoma and normal colon samples were obtained from patients undergoing surgery for colorectal carcinoma (CRC) at two University Hospitals (Second University of Naples and University “Federico II”). Following surgical resection of the bowel tract, a fragment of the tumor and non-pathological tissue were collected from the fresh specimen. Then, samples were divided into several fragments for future extractions of nucleic acids (DNA and RNA) and proteins. Fractionated tissue samples for extractions were then stored at -80 °C.

### Statistical analysis

Statistical analyses were performed through using GraphPad Prism. The comparison between two groups of experiments was carried out using Student’s *t* test. Results are reported as means ± SD and differences were considered to be significant with *p* < 0.05.

## Results

### MiRNA expression profile of MEFs isolated from *Cbx*7-knockout (*Cbx7* KO) mice

First, we analyzed by the miRNACHIP microarray [22] the expression profile of miRNAs in MEFs deriving from WT and *Cbx7* KO mice to identify the miRNAs differentially expressed in MEF null for *Cbx7* in comparison with the WT counterpart. Then, we applied biostatistical analysis (see Methods section), acquiring a list of miRNAs that were differentially expressed (*P* < 0.05) between WT and homozygous *Cbx7* KO MEFs (Tables 1 and 2).

**Table 1:** List of the most studied and up-regulated miRNAs in WT MEFs (cbx7^+/+^) vs KO MEFs (cbx7^-/-^)

Unique Id	Geom. Mean of intensities (cbx7 ^+/+^)	Geom. Mean of intensities (cbx7 ^-/-)^	Ratio of geom Means (cbx7 ^+/+^vs cbx7 ^-/-^)	Parametric *p*-value
Mmu-mir-705	626.5	205.8	3.044	0.0062209
Mmu-mir-221	4233.9	2449.4	1.729	0.0035147
Mmu-mir-222	12079.2	6409	1.885	0.0017689
Mmu-mir-715	127.5	37.2	3.427	0.0016821
Mmu-mir-323	1775	633.6	2.801	0.0003329
Mmu-mir-155	2720.1	508.9	5.345	7.11e-05

**Table 2:** List of the most studied and down-regulated miRNAs in WT MEFs (cbx7^+/+^) vs KO MEFs (cbx7^-/-^)

Unique Id	Geom. Mean of intensities: (cbx7 ^+/+^)	Geom. Mean of intensities: (cbx7 ^-/-^)	Ratio of geom Means (cbx7 ^+/+^ *vs* cbx7 ^-/-)^	Parametric *p*-value
Mmu-mir-137	113.9	687.8	0.166	0.0092591
Mmu-mir-181a-5p	1124.6	2092.2	0.538	0.0030335
Mmu-miR-719	18	186.1	0.097	0.0002231
Mmu-mir-199-5p	445.9	1481.1	0.301	0.0001483

**Fig. 1:**
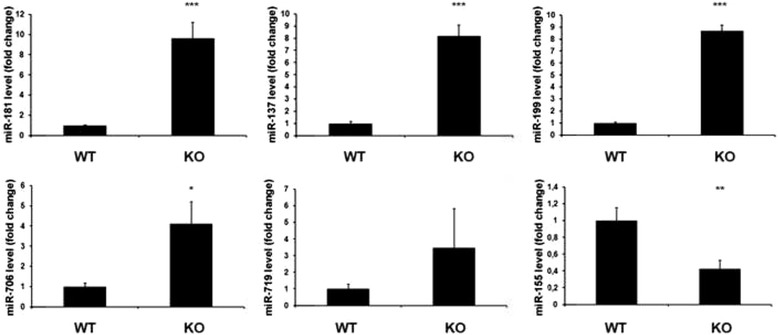
Validation of MiRNA microarray data by qRT-PCR. qRT-PCR analysis of miR-181, miR-137, miR-199, miR-706, miR-719 and miR-155 in mouse embryonic fibroblasts (MEFs) from *Cbx7* ^*-/-*^ (KO) mice compared to wild type (WT), set equal to 1. qRT-PCR analysis was performed in triplicate and reported values represent the mean ± SD. *, *P* < 0.05 **, *P* < 0.01 ***, *P* < 0.001 (*t* test)

The next step was to validate the data acquired by microarray Chip, analyzing 5 deregulated miRNAs by qRT-PCR. The results presented in Fig. 1 confirm a drastic overexpression of miR-181, miR-137, miR-199, miR-706 and miR-719 and repression of miR-155 in *Cbx7* KO MEFs in comparison with the WT ones.

**Fig. 2:**
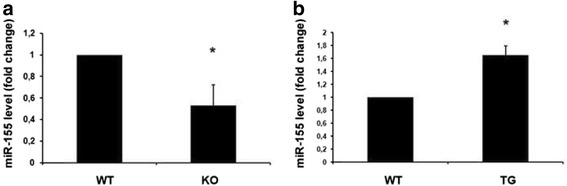
MiR-155 espression in MEFs derived from *Cbx7* null and *Cbx7* transgenic mice. **a** qRT-PCR analysis of miR-155 in MEFs derived from three *Cbx7* knock out lines (KO1, KO2, KO3) and control WT mice and **b** in MEFs derived from *Cbx7* transgenic lines (TG1, TG2, TG3). qRT-PCR analyses were performed in triplicate and reported values represent the mean ± SD. *, *P* < 0.05 (*t* test)

Then, we focused on the miR-155, which revealed the most decreased fold-change in *Cbx7*-null MEFs (Table 1). Moreover, its expression has been frequently found deregulated in human malignancies [26, 27], and its oncogenic activity has been described also in vivo [28]. We then extended the analysis of miR 155 expression, by qRT-PCR, also to MEFs overexpressing *Cbx7* [8] and other *Cbx7* KO MEF preparations. As presented in the Fig. 2a and b, miR-155 levels decrease in KO MEFs in comparison with the WT ones and it was upregulated in those overexpressing *Cbx7*.

### *KRAS* oncogene is target of miR-155

To identify possible mRNA targets of the CBX7-regulated miRNAs, we referred to different on-line available bioinformatic tools (see Methods). Among the numerous potential targets of the miR-155, we focused on *KRAS* because of its key role in development and cancer. Indeed, mutations in the *KRAS* gene have been recurrently unveiled in numerous neoplasias comprising lung, pancreatic and colon carcinomas [29–31], where we have previously found a significant downregulation of CBX7 expression. Moreover, it has also been reported an increased KRAS expression in human colon carcinomas [32].

**Fig. 3:**
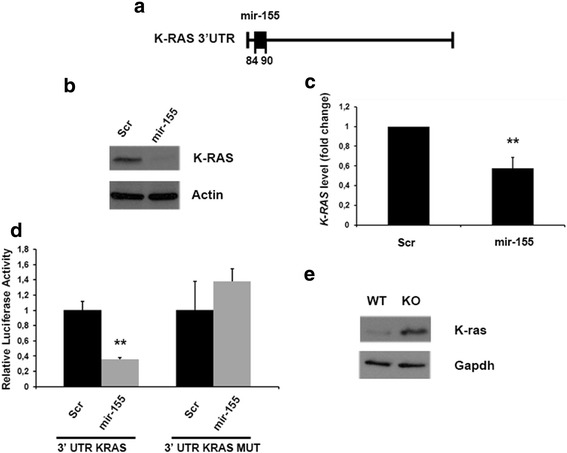
MiR-155 targets KRAS. **a** Schematic representation of *KRAS* 3’ UTR and relative position of the predicted miRNA- binding site. **b** Western blot analysis of KRAS protein levels in HEK-293 cells transfected with miR-155 or a scrambled oligonucleotide. Actin expression was analyzed as loading control. A representative experiment is shown. **c** qRT-PCR analyses of *KRAS* mRNA expression in HEK-293 cells transfected with miR-155 or a scrambled oligonucleotide, normalized with G6PD. qRT-PCR analysis was performed in triplicate and reported values represent the mean ± SD. **d** Relative luciferase activity in HEK-293 cells transiently transfected with 3’UTR-*KRAS* and 3’UTR-*KRAS* mutated in the miR-155 seed sequence along with the miR 155 or with a scrambled oligonuclotide. **e** Western blot analysis of KRAS protein in MEFs Cbx7^+/+^ (WT) and Cbx7^-/-^ (KO). Gapdh expression was analyzed as loading control. **, *P* < 0.01 (*t* test)

In Fig. 3 Panel a, we show that 3’UTR of *KRAS* has one putative binding site for the miR-155. Then, to confirm the effect of the miR-155 on the selected candidate target, we evaluated KRAS protein levels in 293 cells transfected with the miR-155, by western blot analysis. As presented in Fig. 3b, introduction of the miR-155 critically decreases the KRAS protein levels. Conversely, no variations in KRAS protein levels were found when the cells were transfected with a scrambled oligonucleotide. Interestingly, modifications in the *KRAS* mRNA amounts were detected in the cells transfected with the miR-155 (Fig. 3c), thus indicating a role of miR-155 in *KRAS* mRNA degradation.

Several miRNAs regulate gene expression through base pairing with the miRNA-recognizing elements (miR-RE) in their mRNA target. To establish whether the direct interaction between the miR-155 and *KRAS* mRNA is responsible for reduced expression of this protein, we introduced downstream of the luciferase ORF, 485 bp (25-510 bp) of the 3’-UTR of the *KRAS* mRNA. 293 cells were transfected with the generated reporter vector and the miR-155. Luciferase activity was much lower after miR-155 transfection (Fig. 3d) than after transfection with the scrambled oligonucleotide. The same reporter construct of the previous experiments, but carrying mutations in *KRAS* 3’ UTR at the miR-155 target site, was unresponsive to the effects of miR-155 (Fig. 3d), proving that the modifications of the target site of *KRAS* 3’ UTR are able to block the function of this miRNA.

In agreement with the capacity of miR-155 to target *KRAS* mRNA, the relative protein amounts are higher in *cbx7* KO MEFs in comparison with the WT (Fig. 3e).

**Fig. 4:**
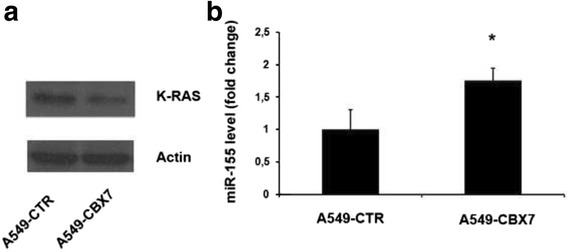
KRAS and miR-155 espression in A549 cell clones transfected with CBX7. **a**Western blot analysis of KRAS protein in A549 cells expressing CBX7 (A549-CBX7) *versus* A549 cells transfected with the empty vector (A549-CTR). Actin expression was analyzed as loading control. **b** qRT-PCR analysis of miR-155 expression in A549-CBX7 cells *versus* A549-CTR cells, normalized with G6PD. qRT-PCR analysis was performed in triplicate and reported values represent the mean ± SD. *, *P* < 0.05 (*t* test)

According to the results obtained on *cbx7* null MEFs, the levels of KRAS protein were found downregulated in A549 cells (human lung carcinoma line), overexpressing the CBX7 protein (Fig. 4a), in which we found higher miR-155 levels with respect to the same cells transfected with the control vector (Fig. 4b).

### miR-155 expression positively correlates with *CBX7* in human colon carcinoma

**Fig. 5:**
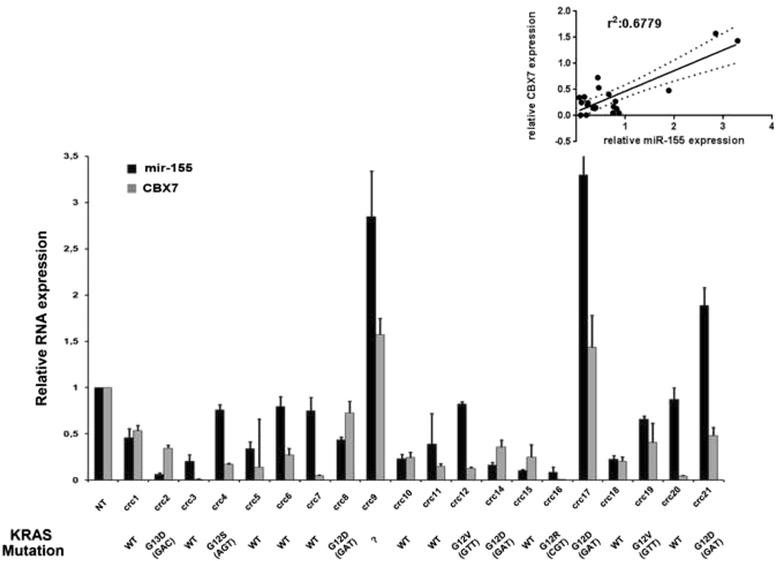
Correlation between CBX7 expression and miR-155 expression in colon carcinoma tissues. qRT-PCR analysis of *CBX7* and miR-155 expression in colorectal carcinoma (CRC) compared with normal tissue. qRT-PCR analysis was performed in triplicate and reported values represent the mean ± SD. The fold change indicates the relative change in expression levels between tumors samples and normal samples, assuming that the value of each normal sample is equal to 1. Linear regressions of *CBX7* versus mir-155 is shown. KRAS mutation status of the colon cancer samples are indicated at the bottom of the figure

Then, we have evaluated miR-155 and the *CBX7* gene expression in colon carcinoma tissues by qRT-PCR. As presented in Fig. 5, downregulation of both miR-155 and *CBX7* was observed in most of the carcinoma samples in comparison with the normal colon mucosa. Linear regression between *CBX7* and miR-155 expression is also reported (Fig. 5).

The direct correlation between *CBX7* and miR-155 expression suggests that these genes are co-regulated in human colon carcinomas. The presence of a mutated KRAs status does seem to have any correlation with miR-155 or *CBX7* expression.

We have also analyzed *KRAS* expression at mRNA level. These results are shown in the Additional file 1: Figure S1. There is no significant correlation with *CBX7* and miR-155 levels. It is likely that several other factors act on *KRAS* mRNA levels in vivo, and maybe the effect of miR-155 is exerted particularly at translation level.

## Discussion

*CBX7* has been already proposed as tumor suppressor gene, since several studies have demonstrated that its expression is drastically downregulated in most of the malignant neoplasias [4], and the development of liver and lung carcinomas in *Cbx-7* null mice further supports the CBX7 tumor suppressor activity [8]. Moreover, the restoration of *CBX7* expression in carcinoma cells of different origin results into a reduced growth rate [5, 7, 11, 33], blocking the cells in the G1 phase of the cell cycle. Very recently, we have also reported that reestablishment of *CBX7* expression in two cell lines of human lung carcinoma, in which it was undetectable, yields a diminished proliferation and an improved apoptosis after drug exposure [21]. However, CBX7 has been reported to exert also oncogenic activity. Indeed, it was shown that *Cbx7* expression in mice lymphoid compartment can promote T cell lymphomagenesis and, working together with c-Myc, produces aggressive B cell lymphomas by downregulating Ink4a/Arf locus [1]. Equally, CBX7-positive patients affected by ovarian carcinoma showed significantly shorter overall and progression-free survival rates than those of the CBX7-negative patients. Moreover, *CBX7* knockdown significantly reduced cell viability in two ovarian carcinoma cell lines compared to the control cells, likely by the upregulation of tumor necrosis factor-related apoptosis-inducing ligand (TRAIL) [34]. Recently, we have reported that CBX7 regulates several genes involved in tumor progression [12, 35] such as E cadherin, cyclin E, *SPP1* likely accounting for the critical role of CBX7 in carcinogenesis.

In this study, we have envisaged the hypothesis that CBX7 tumor suppressor or oncogenic role may be also mediated by the regulation of miRNA expression since the role of miRNAs in cancer development and progression has been frequently reported [36]. Consistently, we previously demonstrated that CBX7 negatively regulates the expression of miR-181 that has among its targets CBX7, creating a synergistic loop that contributes to breast cancer progression [11].

Then, we have studied the miRNA expression profile of *Cbx7* null MEFs *versus* the wt. Nine miRNAs were found downregulated and twenty upregulated with a fold change higher than 2 in the *Cbx7* KO MEFs with respect to WT counterparts. We first validated the results of the array analyzing, by qRT-PCR, 5 deregulated miRNAs in *Cbx7* KO and WT MEFs.

On the basis of the opposite functions of CBX7, as oncogene and oncosuppressor, it was not surprising to find that CBX7 was able to regulate in opposite sense miRNAs that have recognized to have oncogenic functions, such as miR-199 (negatively regulated) and miR-155, miR-221 and miR-222 (positively regulated). Equally, we observe that a potential oncosuppressor gene, such as miR-137, is regulated negatively, whereas another one, such as miR-323, is regulated positively. It is also not unlikely that the modulation of these miRNAs might be specific of MEFs and that CBX7 could regulate the same miRNA positively or negatively depending on the cellular context. Therefore, we retain that the recent results evidencing a role of the loss of CBX7 expression in the progression of human colon cancer might be also explained by the ability of CBX7 to modulate the expression of several miRNAs.

Subsequently, we decided to focus on the miR-155 since it was one of the most downregulated miRNAs in *Cbx7* KO MEFs. Moreover, miR-155 is the most commonly miRNA overexpressed in malignancies among the cancer-related miRNAs [28]. Bcl6, HDAC4, msh2, msh6, mlh1 have been already identified as targets of miR-155 [26]. In liposarcoma casein kinase 1-α (CK1-α) is targeted by miR-155, enhancing beta-catenin and cyclin D1 [27]. In this study we have identified *KRAS* as target of miR-155, since overexpression of miR-155 leads to a drastic reduction of the *KRAS* mRNA and protein levels indicating an effect of miR-155 also on *KRAS* mRNA degradation. Consistently, higher KRAS protein levels were detected in *Cbx7* null MEFs in comparison with the WT. The same result was achieved when *CBX7* expression was restored in lung carcinoma cells. However, the analysis of KRAS specific mRNA does not reveal any correlation with miR-155 and CBX7 expression. Maybe this correlation is present at protein levels, and we can also hypothesize that the regulation of KRAS by miR-155 is specific of some tissues depending on the cellular context.

It is likely that the targeting of *KRAS* may have a role in the anti-apoptotic activity of miR-155 observed in monocytic differentiation where miR-155 seems to have as targets other anti-apoptotic factors such as RPS6KA3, SGK3, and RHEB. It is likely that this anti-apoptotic activity may be important for the growth of MEFs, also associated to the increased mR-221 and miR-222 levels that are able to target p27 [37], a critical negative regulator of the cell cycle.

Interestingly, CBX7 seems to positively regulate miR-323: this can account for the reduced miR-323 expression in prostate cancer where CBX7 is drastically downregulated [38] and miR-323 has a tumor suppressor activity by targeting AdipoRI [39].

## Conclusion

In conclusion, these studies demonstrate that CBX7 is able to positively regulate miR-155 and identify KRAS as one of the target genes of this miRNA likely accounting for the anti-apoptotic activity ascribed to miR-155 in some tissue context.

## Additional file

Additional file 1:
**Figure S1.** qRT-PCR analysis of KRAS expression in colorectal carcinoma (CRC) compared with normal tissue. qRT-PCR analysis was performed in triplicate and reported values represent the mean ± SD. The fold change indicates the relative change in expression levels between tumors samples and normal samples, assuming that the value of each normal sample is equal to 1. (TIF 757 kb)

## Abbreviations

HEK: Human embryonic kidney 293 cells
KO: Knockout
MEF: Mouse embryonic fibroblasts
miRNA: microRNA
PRC: Polycomb repressive complex
qRT-PCR: Quantitative real time PCR
TG: Transgenic
WT: Wild-type
