# Supplementary figures and images for: miR-155 is positively regulated by CBX7 in mouse embryonic fibroblasts and colon carcinomas, and targets the KRAS oncogene
Source: BMC Cancer. 2017 Mar 4;17:170. doi: 10.1186/s12885-017-3158-z (PMC5336640; doi:10.1186/s12885-017-3158-z)

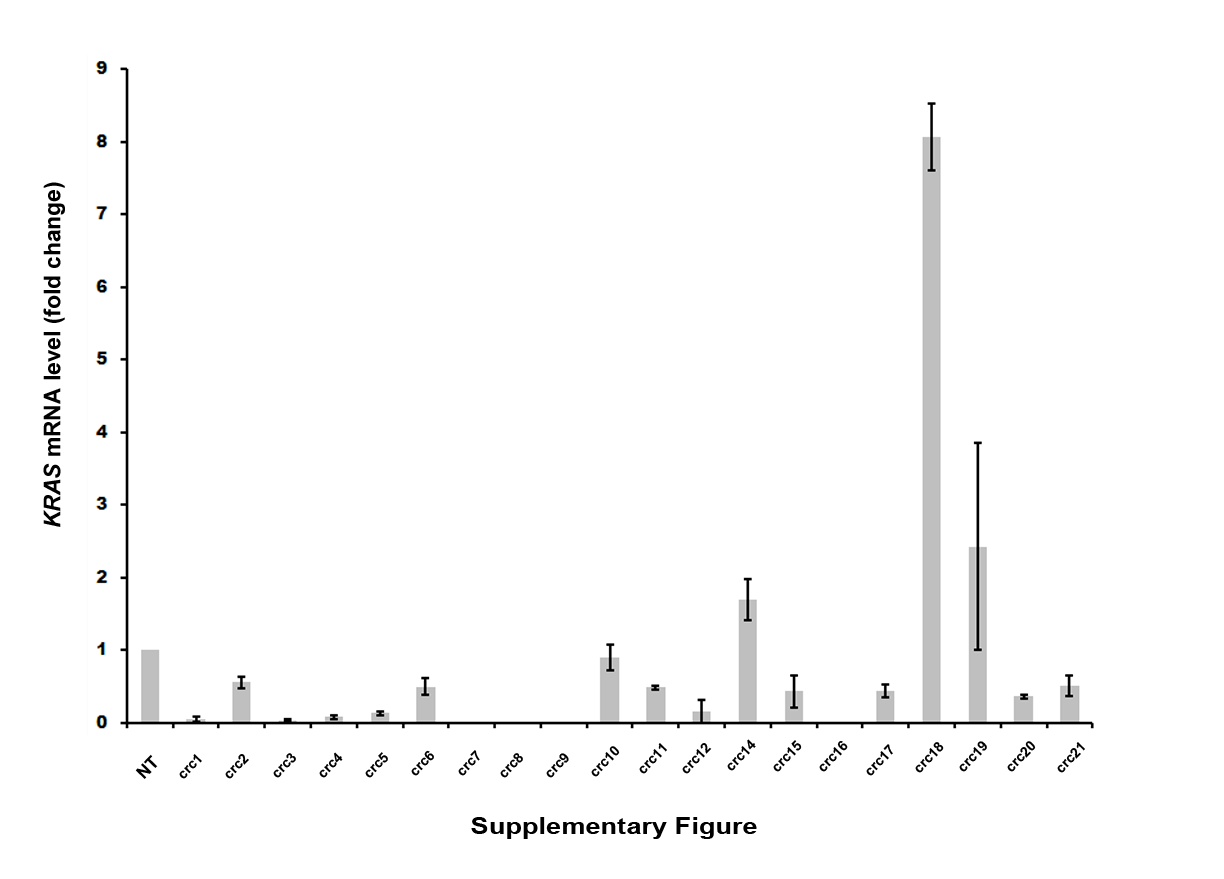

Supplement: Additional file 1: — Figure S1. qRT-PCR analysis of KRAS expression in colorectal carcinoma (CRC) compared with normal tissue. qRT-PCR analysis was performed in triplicate and reported values represent the mean ± SD. The fold change indicates the relative change in expression levels between tumors samples and normal samples, assuming that the value of each normal sample is equal to 1. (TIF 757 kb) [file 12885_2017_3158_MOESM1_ESM.tif]
